# SARS-CoV-2 vaccination and multiple sclerosis: a large multicentric study on relapse risk after the third booster dose

**DOI:** 10.1007/s00415-023-12034-0

**Published:** 2023-11-03

**Authors:** Massimiliano Di Filippo, Diana Ferraro, Paolo Ragonese, Luca Prosperini, Giorgia Teresa Maniscalco, Antonio Gallo, Paola Cavalla, Lorena Lorefice, Viviana Nociti, Elena Di Sabatino, Marinella Clerico, Clara Guaschino, Marta Radaelli, Roberta Fantozzi, Fabio Buttari, Alice Laroni, Alberto Gajofatto, Massimiliano Calabrese, Simona Malucchi, Damiano Paolicelli, Giovanna De Luca, Valentina Tomassini, Roberta Lanzillo, Marcello Moccia, Claudio Solaro, Eleonora Cocco, Claudio Gasperini, Carla Tortorella, the RIREMS (Rising Researchers in MS) group

**Affiliations:** 1 Clinica Neurologica, Dipartimento di Medicina e Chirurgia, Università di Perugia, Perugia, Umbria Italy; 2 Dipartimento di Neuroscienze, Ospedale Civile di Baggiovara, Azienda Ospedaliera-Università di Modena, Modena, Italy; 3 Dipartimento di Biomedicina, Neuroscienze e Diagnostica Avanzata, Università degli Studi di Palermo, Palermo, Italy; 4 Dipartimento di Neuroscienze, Ospedale San Camillo-Forlanini, Rome, Italy; 5 Centro Regionale Sclerosi Multipla, Dipartimento di Neurologia e Stroke Unit, Ospedale “A. Cardarelli”, Naples, Italy; 6 Dipartimento di Scienze Mediche e Chirurgiche Avanzate, Università degli Studi Della Campania Luigi Vanvitelli, Naples, Italy; 7 Centro Sclerosi Multipla e Neurologia 1 D.U, Dipartimento di Neuroscienze e Salute Mentale, Azienda Ospedaliero-Universitaria Città della Salute e della Scienza di Torino, Turin, Italy; 8 Centro Sclerosi Multipla, Dipartimento di Scienze Mediche e di Sanità Pubblica, ASL Cagliari, Università di Cagliari, Ospedale Binaghi, Cagliari, Italy; 9 Centro Sclerosi Multipla, Fondazione Policlinico Universitario Agostino Gemelli IRCCS, Università Cattolica del Sacro Cuore, Rome, Italy; 10 Dipartimento di Scienze Cliniche e Biologiche dell’Università di Torino, AOU San Luigi Gonzaga Di Orbassano, Orbassano, Italy; 11 Neurologia ad Indirizzo Neuroimmunologico-Centro Sclerosi Multipla, ASST Valle Olona, Gallarate, Italy; 12 U.O.C. Di Neurologia, ASST Papa Giovanni XXIII, Bergamo, Italy; 13 Unità Di Neurologia, IRCCS Neuromed, Pozzilli, Italy; 14 Dipartimento di Medicina dei Sistemi, Università di Tor Vergata, Rome, Italy; 15 Dipartimento di Neuroscienze, Riabilitazione, Oftalmologia, Genetica, Scienze Materno-Infantili, Università di Genova, Genoa, Italy; 16 IRCCS Ospedale Policlinico San Martino, Genoa, Italy; 17 Dipartimento di Neuroscienze, Biomedicina e Movimento, Università di Verona, Verona, Italy; 18 SCDO Neurologia – CRESM, AOU San Luigi Gonzaga, Orbassano, Italy; 19 Dipartimento di Biomedicina Traslazionale e Neuroscienze, Università degli Studi di Bari Aldo Moro, Bari, Italy; 20 Centro Sclerosi Multipla, Clinica Neurologica, Ospedale Universitario SS Annunziata, Chieti, Italy; 21 Istituto di Tecnologie Avanzate Biomediche (ITAB), Dipartimento di Neuroscienze, Imaging e Scienze Cliniche, Facoltà di Medicina e Chirurgia, Università di Chieti-Pescara “G. D’Annunzio”, Chieti, Italy; 22 Dipartimento di Neuroscienze e Scienze Riproduttive ed Odontostomatologiche, Università Degli Studi Federico II, Naples, Italy; 23 Dipartimento di Medicina Molecolare e Biotecnologie Mediche, Università degli Studi di Napoli Federico II, Naples, Italy; 24 Centro Sclerosi Multipla, Ospedale Universitario Federico II, Naples, Italy; 25 Unità di Neurologia, E.O. Ospedali Galliera, Genoa, Italy; 26 Dipartimento di Scienze Mediche e Sanità Pubblica, Centro Regionale Sclerosi Multipla, ASL Cagliari, ATS Sardegna, Università di Cagliari, Cagliari, Italy

**Keywords:** COVID-19, SARS-CoV-2, Vaccination, Multiple sclerosis

## Abstract

**Background:**

COVID-19 vaccines have been recommended to people with multiple sclerosis (pwMS) and, to ensure durable immunity, a third booster dose has been administered in several countries. Data about potential risks associated with the third booster dose in pwMS, such as vaccine-triggered disease exacerbations, are still scarce.

**Objective:**

To investigate whether the administration of a third booster dose of mRNA COVID-19 vaccines was associated with an increased risk of short-term disease reactivation in a large cohort of pwMS.

**Methods:**

We retrospectively selected 1265 pwMS who received a third booster dose of an mRNA COVID-19 vaccine. Demographic and clinical data were collected, including the presence, number and characteristics of relapses in the 60 days prior to and after the third booster dose.

**Results:**

In the selected cohort, the relapse rate in the two months after administration of the third booster dose of mRNA COVID-19 vaccines did not increase when compared with the prior two months. Indeed, the percentage of pwMS experiencing relapses in the 60 days following the administration of the third booster dose was 2.1%, similar to the percentage recorded in 60 days prior to vaccination, which was 1.9%.

**Conclusions:**

The third booster dose of mRNA COVID-19 vaccines appeared to be safe for pwMS.

## Introduction

The severe acute respiratory syndrome coronavirus 2 (SARS-CoV-2) pandemic has represented a global emergency with rapid spread, significant morbidity and mortality, as well as substantial health and socio-economic consequences [1]. In this scenario, vaccination has constituted the most promising path back to ‘normal life’ [2–4]. Nonetheless, the large-scale application of recently approved vaccines has raised concerns regarding their safety, re-igniting the long-standing debate on autoimmunity and vaccines. The fear of adverse effects of COVID-19 vaccines is especially pronounced in people suffering from chronic autoimmune diseases, such as multiple sclerosis (MS), motivated by the risk of aberrant immune-mediated responses triggered by the vaccination [5–7].

Several national and international MS societies have recommended COVID-19 vaccination in MS, given the incidence of COVID-19 and a more severe course of the disease, especially among most fragile people with MS (pwMS) [8]. On the other hand, pwMS have not been included in randomized controlled clinical trials for the approval of currently licensed vaccines. Hence, real-life data on the safety of the COVID-19 vaccines are of utmost importance. The so-far available safety evidence in pwMS has been mainly derived from observational studies that have produced reassuring results regarding the potential for vaccination to drive pathogenic immune responses triggering disease reactivation and/or other relevant adverse events. Reported adverse events are generally limited to local reactions and mild systemic reactions such as fever [9–15]. Alongside, however, several case-reports and case-series on the occurrence of MS relapses after SARS-CoV-2 vaccination have been published and there is still a considerable uncertainty among pwMS regarding COVID-19 vaccines [16–19]. Meanwhile, the landscape of SARS-CoV-2 vaccinology is rapidly evolving, thereby necessitating continuous monitoring. Additionally, a waning of the humoral immune response within six months after the initial immunization has been reported, particularly in specific subpopulations such as pwMS who are on ocrelizumab or fingolimod treatments [20, 21]. Therefore, the administration of a third booster dose has been approved and performed in several countries [22, 23]. However, since pwMS constitute a rather unique population regarding immune responses, the safety profile of the booster vaccine, which represents an additional stimulus to an immune system formerly activated by the first immunization cycle, must be thoroughly assessed. This becomes even more important in the context of the only limited literature pertaining to the safety of the third booster dose [24–26].

In this retrospective study, we aimed to investigate the adverse event profile of a third booster dose of mRNA COVID-19 vaccines in a large cohort of pwMS. Specifically, we evaluated whether the third dose was associated with an increased incidence of disease relapses within the two months after the booster dose compared to the prior two months. Selecting a two-month interval following the third mRNA COVID-19 vaccine booster was driven by the typical short timeframe, ranging from few weeks to a couple of months, in which neurological autoimmune reactions triggered by vaccinations tend to develop [5].

## Patients and methods

### Patients’ selection

Twenty Italian MS tertiary centres participated to this self-controlled, multicentric, observational study. We retrospectively selected consecutive pwMS who underwent an outpatient visit at each participating centre performed in the period between April 2022 and May 2022. Eligible subjects were identified within the MS registry of each centre according to the following inclusion criteria: (1) age ≥ 18 years; (2) a diagnosis of relapsing remitting MS (RRMS) or progressive MS (PMS) according to the 2017 revision of the McDonald criteria [27]; (3) having received a third booster dose of an mRNA COVID-19 vaccine, at least 6 months apart from the second dose according to national recommendation.

### Data collection

All the demographic and clinical data were anonymously collected in an electronic database. The database was locked on 06 December 2022. The following data were recorded: (1) sex; (2) age and disease duration; (3) disease course (relapsing remitting; progressive); (4) date of the first, second and third vaccine doses administration; (5) disability score (as measured by the Expanded Disability Status Scale, EDSS) at the time of the third booster dose; (6) disease modifying treatments (DMTs) at the time of the third booster dose; (7) clinical relapses in the year before third booster dose, with specific regard to the 2 months immediately preceding vaccination; (8) previous influenza vaccine administration. The presence, characteristics and number of relapses occurring in the 60 days after the third booster dose were recorded. A relapse was defined as a clinical episode suggestive of demyelination developing acutely or subacutely (e.g., diplopia, difficulty in walking, numbness), with a duration of at least 24 h in the absence of fever or infection [27]. The interval between vaccination with the third booster dose and clinical relapse was calculated. Approval number of the ethics committee of the coordinating center (CER Umbria): 4315/19.

### Statistical analysis

Continuous variables are reported as mean ± standard deviation (SD) or median (interquartile range [IQR]), while categorical variables are reported as count and percentages. For the assessment of the short-term risk of post-vaccine disease reactivation, we compared the percentage of pwMS who experienced relapses in the two months period before and after the third booster dose via McNemar paired test. Significance threshold was set at p value < 0.05. Statistical analysis was performed with SPSS Statistics, version 25.

## Results

We included 1265 pwMS, 66,6% of whom were females, with a mean age of 44.4 years, exposed to a third booster dose of an mRNA COVID19 vaccine. Overall, the majority of the cohort exhibited the RR phenotype (84.4%) and was receiving a DMT at the time of the third booster dose (89.5%). Among the 197 (15.6%) individuals included with PMS, 15 (7.6%) exhibited relapses in the previous year. The average time between the second vaccine dose and the third booster dose was of 7.4 ± 1.3 months. Detailed cohort’s characteristics are reported in Table 1.

**Table 1:** Cohort’s characteristics

Characteristics of the cohort	
Demographic features	
Number	1265
Age – yrs; mean ± SD	44.4 ± 12
Female; n (%)	842 (66.6%)
Disease phenotypes; n (%)	
RR	1068 (84.4%)
P	197 (15.6%)
Disease characteristics at the time of 3rd dose	
Disease duration –yrs; median (IQR)	11.2 (14)
EDSS; mean ± SD	2.6 ± 2
Disease activity in the year before 3^rd^ dose	
Number of pwMS with relapses; n (%)	157 (12.4%)
DMTs at the time of 3^rd^ dose; n (%)	
Glatiramer acetate	80 (6.3%)
Interferons	127 (10%)
Teriflunomide	66 (5.2%)
Dimethyl fumarate	207(16.4%)
Fingolimod	153 (12.1%)
Ozanimod	2 (0.2%)
Siponimod	11 (0.9%)
Cladribine	42 (3.3%)
Natalizumab	227 (17.9%)
Ocrelizumab	178 (14.1%)
Ofatumumab	1 (0.1%)
Rituximab	7 (0.6%)
Azathioprine	11 (0.9%)
Alemtuzumab	19 (1.5%)
Bone marrow transplant	1 (0.1%)
No therapy	133 (10.5%)
Vaccine type; n (%)	
Pfizer/BNT162b2 mRNA COVID-19 vaccine	1082 (85.5%)
Moderna/COVID-19 mRNA-1273	183 (14.5%)
Distance 2nd dose–3rd dose COVID-19 vaccine – months; mean ± SD	7.4 ± 1.3
Influenza vaccine before 3rd dose	
Yes; n (%)	99 (7.8.%)
Distance influenza vaccine-3rd dose—months; median (IQR)	4.8 (27.1)

Data are expressed as number (percentage), mean ± standard deviation or median (range)

*pwMS* people with MS, *yrs* years, *RR* relapsing–remitting, *P* progressive, *EDSS* expanded disability status scale, *DMTs* disease modifying treatments

In the year prior to third booster dose, 174 relapses were recorded in 157 out of 1265 pwMS (12.4%). In the 2 months prior to third booster dose vaccination, 24 clinical relapses were reported in 24 out of 1265 pwMS (1.9%). In the 2 months after the vaccination, 28 clinical relapses occurred in 27 out of 1265 pwMS (2.1%). The percentage of pwMS with relapses in the two-month period before and after vaccination was not statistically significant (p > 0.05). Fifteen out of the 27 (55.6%) pwMS who relapsed in the two months following vaccination were women and 25/27 (92.6%) presented RRMS. Both the two individuals with PMS who relapsed in the 60 days following the third booster dose did not experience clinical disease activity in the year prior to vaccine. Twenty-one relapses were monofocal and 7 were multifocal. The mean time interval between the third booster dose administration and clinical relapse was 31.6 ± 20.6 days. At the time of vaccination, 8/27 (29.6%) pwMS who experienced a relapse were not treated with DMTs.

## Discussion

Accumulating evidence suggests that the benefits of SARS-CoV-2 vaccines in pwMS outweigh potential vaccine-related risks. Nevertheless, vaccine hesitancy among pwMS remains an issue [28]. This work provides evidence that the third booster dose of an mRNA COVID-19 vaccine does not increase the short-term risk of clinical reactivation in pwMS. We have already provided safety information related to COVID-19 vaccines in another observational study, with a similar design, on 324 pwMS after the first immunization cycle [11]. Interestingly, in the current study, we found that the rate of pwMS with acute relapses following the third booster dose vaccination was similar to the rate we have reported following the administration of the first mRNA COVID-19 vaccine cycle (i.e., 2.1 vs 2.2%) [11]. Conversely, a recent large-scale retrospective study, with a different design, involving 1661 pwMS found that there was a mild increase in the proportion of pwMS experiencing at least one clinical relapse in the 90 days after the first COVID-19 vaccination compared to the 90-days intervals during the year before [29]. Another prospective, observational study on a large cohort from the German MS Registry (GMSR) conducted by Frahm and colleague found that, during a median observation period of 4.5 months following vaccination, relapses occurred in 245 out of 2661 pwMS (9.3%; extrapolated annualized relapse rate [ARR] of 0.19). A comparison of ARRs one year before and after vaccination in a reference cohort from the GMSR indicated no substantially increased relapse activity after SARS-CoV-2 vaccination in pwMS [30]. Furthermore, our finding is supported by a systemic review and meta-analysis including data from 14755 pwMS who received a cumulative number of 23088 doses of any SARS-CoV-2 vaccine (i.e., mRNA, inactivated virus and adeno-vector), which documented a pooled proportion of pwMS experiencing relapses at an average time interval of 20 days from SARS- CoV-2 vaccination of 1.9% [31].

The main result of the study is also aligned with previous, albeit limited, literature concerning the risk of disease reactivation following the third booster dose, and confirms an overall favorable safety profile of vaccination in pwMS. For instance, in two recent observational prospective studies, on 130 and 47 pwMS respectively, treated with an anti-CD20 therapy or fingolimod, no relapses were recorded after revaccination. The latter studies, however, suffer the potential bias due to the recruitment of subjects undergoing specific pharmacological interventions, since the inclusion of pwMS under highly effective DMTs might have lowered the number of recorded relapses [24, 25]. Another study on a cohort of 211 fully vaccinated pwMS reported that within the first 30 days following the third vaccine dose, 1.4% of the study population presented a relapse, with a rate that raised at 3.3% after a median follow-up of 66 days [26]. Notably, none of these studies compared the incidence of relapses prior to and after vaccination. Regarding the long-term safety assessment of the booster dose of anti-SARS-CoV-2 vaccines, in a monocentric Italian study on a cohort of 114 pwMS with a median follow-up of 6 months post-booster dose, MS relapses were observed in four cases (3.5%), two of which occurring within 8 weeks after the booster dose (1.7%) [32]. However, it's important to highlight that the absence of a control population makes it challenging to draw definitive conclusions from these data. Finally, our findings are consistent with the results from large case–crossover studies (i.e., comparing vaccinated to unvaccinated pwMS) and meta-analyses, that have reported no excess relapse risk during the post-vaccination period, for vaccines including tetanus, hepatitis B, Bacille Calmette–Guèrin (BCG), and influenza [33].

In conclusion our study is the first study including a large cohort of pwMS who were followed, with a self-controlled design, for 2 months after receiving the third booster dose of an mRNA COVID-19 vaccine. Moreover, the study population is well-characterized and includes both people with RRMS and with PMS under any DMTs or untreated; this comprehensive representation closely mirrors real-world scenarios and clinical-demographic diversity. The larger cohort size and the focus on the third booster dose, which implies repeated immune system stimulation, strengthened the findings from our previous study [11]. However, for an accurate interpretation of our results, certain limitations must be acknowledged. First, selection bias could be present, since pwMS receiving the third booster dose of COVID-19 vaccine are likely those who did not experience relapses or severe adverse events after initial immunization. Furthermore, the mean age of pwMS included is quite advanced (44.4 years with a median disease duration of 11.2 years), an age range potentially associated with lower disease activity. It is worth noting that a significant portion of the cohort was under DMTs, this limiting the potential generalization of the results to untreated pwMS. Another limit of our study, mainly related to its real-life context, is the lack of magnetic resonance imaging (MRI) data, that could have allowed the detection of potential MRI activity in absence of clinical relapses. Future studies including MRI data and with longer follow-up times could be useful in this scenario.

Despite these limitations, results of our study support the idea that COVID-19 vaccination usefulness outweighs its potential risks in pwMS. In the age of vaccination skepticism, it is of paramount importance to reiterate that numerous epidemiological studies have not found evidence of MS reactivation being triggered by vaccines. On the contrary, in weighing the potential advantages and disadvantages of vaccines, it is noteworthy that vaccines can prevent some infections known to increase the risk of relapses [34] and, consequently, long-term disability in pwMS.

## Data Availability

Study data are available upon request.
